# Supplementary material for: Rainfall as a driver for near-surface turbulence and air-water gas exchange in freshwater aquatic systems
Source: PLoS One. 2024 Mar 12;19(3):e0299998. doi: 10.1371/journal.pone.0299998 (PMC10931499; doi:10.1371/journal.pone.0299998)
Supplement: S1 Appendix — (PDF) [file pone.0299998.s001.pdf]

S1 Appendix. Estimate of the accuracy of the PIV System

According to Thielicke (2014), the accuracy of the PIV velocity estimates depends mainly on the cross-correlation algorithm and the choice of the peak-finding technique. By using the DFT plus deformation window approach and running several passes, the error sources, such as loss of information and background noise in the correlation matrix, are significantly mitigated. The Gaussian 2.3 –point fit for the peak finding in the cross correlation refines the results to a subpixel precision, but normally some complications in the detection of the intensity peak can occur due to non-uniform particle motion inside the interrogation area, which broadens the intensity peak. The use of the deformation window reduces shear and rotation within the interrogation area.
The particle diameters and particle density in the interrogation area also influence the accuracy of the velocity measurements. Thielicke (2014) recommends diameters larger than 3 pixels and particle densities up to 20 particles per interrogation area (IA) (at the final pass). Under these conditions and the use of DFT plus deformation window algorithm, and the Gaussian 2.3-point fit for the peak finding, this author found bias errors <0.005 pixels and random errors <0.02 pixels. In our experiment, we used the recommended algorithm and peak-finding settings, the last pass of the deformation area was at 64 × 64 of the IA and we obtained a final particle median particle diameter of 3.9 pixels and a particle density of 12.6 particles per IA (n = 4503).

References

Thielicke W. The Flapping Flight of Birds: Analysis and application. 2014. doi:10.1017/s036839310013768x
