# Supplementary material for: Rainfall as a driver for near-surface turbulence and air-water gas exchange in freshwater aquatic systems
Source: PLoS One. 2024 Mar 12;19(3):e0299998. doi: 10.1371/journal.pone.0299998 (PMC10931499; doi:10.1371/journal.pone.0299998)
Supplement: S1 Table — (PDF) [file pone.0299998.s002.pdf]

S1 Table. Comparison of turbulent dissipation rates from PIV_dir (equation (9)), PIV_spec and ADV (equation (10)) for all runs at the ADV sampling point location (10 cm depth).

R<br>[mm h^{-1}]	ϵ_{PIV_dir}<br>[W/kg]	ϵ_{PIV_spec}<br>[W/kg]	ϵ_{ADV}<br>[W/kg]
6.9	4.82×10^{-8}	1.42×10^{-8}	1.20×10^{-8}
8.1	4.85×10^{-8}	1.29×10^{-8}	1.10×10^{-8}
10.3	9.52×10^{-8}	4.03×10^{-8}	7.50×10^{-8}
13.5	1.61×10^{-7}	8.03×10^{-8}	4.29×10^{-6}
16.0	1.69×10^{-7}	3.06×10^{-8}	2.29×10^{-7}
21.1	3.55×10^{-8}	5.31×10^{-9}	4.99×10^{-7}
16.2	2.59×10^{-7}	1.52×10^{-7}	6.74×10^{-7}
19.8	3.16×10^{-7}	1.13×10^{-6}	1.47×10^{-7}
25.0	9.09×10^{-8}	1.71×10^{-7}	5.77×10^{-7}
26.0	1.88×10^{-7}	1.15×10^{-7}	3.28×10^{-6}
28.8	1.77×10^{-7}	8.36×10^{-7}	4.16×10^{-6}
39.4	3.66×10^{-7}	1.05×10^{-7}	1.33×10^{-6}
49.0	3.03×10^{-7}	1.27×10^{-7}	5.23×10^{-6}
89.0	4.20×10^{-7}	3.06×10^{-7}	2.48×10^{-6}
