# Supplementary material for: Rainfall as a driver for near-surface turbulence and air-water gas exchange in freshwater aquatic systems
Source: PLoS One. 2024 Mar 12;19(3):e0299998. doi: 10.1371/journal.pone.0299998 (PMC10931499; doi:10.1371/journal.pone.0299998)
Supplement: S1 Fig — Pictures of the experimental setup: a) Hose-drying tower (approximately 20 m tall) of the municipal fire brigade in Landau, Germany, in which experiments were conducted. b) Inside view showing the rain generator in the top and the aquarium at the base of the tower. c)detailed view of the rain generator (cf. Fig 1). d) Aquarium with sensors and camera mounting frame. (PDF) [file pone.0299998.s003.pdf]

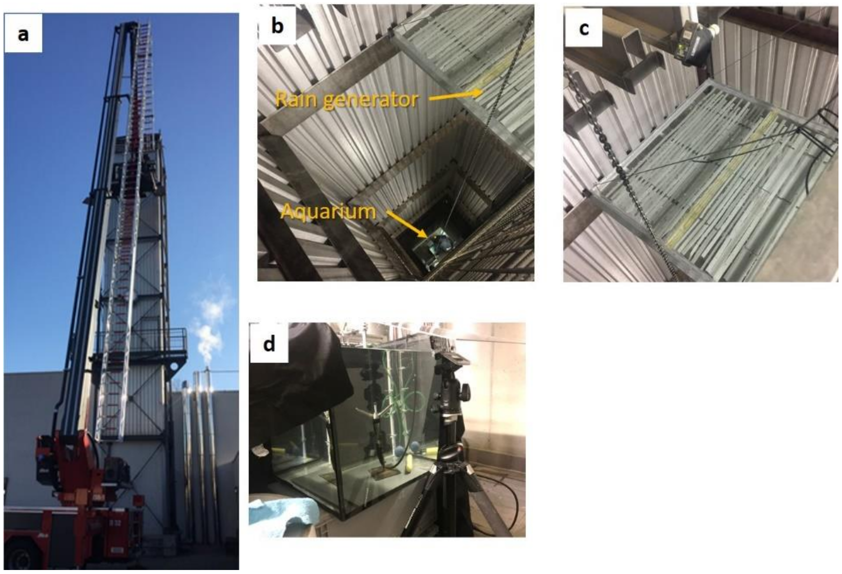

S1 Fig. Pictures of the experimental setup: a) Hose-drying tower (approximately 20 m tall) of the municipal fire brigade in Landau, Germany, in which experiments were conducted. b) Inside view showing the rain generator in the top and the aquarium at the base of the tower. c)detailed view of the rain generator (cf. Fig. 1). d) Aquarium with sensors and camera mounting frame.
