# Supplementary material for: Rainfall as a driver for near-surface turbulence and air-water gas exchange in freshwater aquatic systems
Source: PLoS One. 2024 Mar 12;19(3):e0299998. doi: 10.1371/journal.pone.0299998 (PMC10931499; doi:10.1371/journal.pone.0299998)
Supplement: S2 Fig — The rainfall rate was varied by closing or opening a varying number of holes using adhesive tape (visible as black and silver tape stripes in the picture). (PDF) [file pone.0299998.s004.pdf]

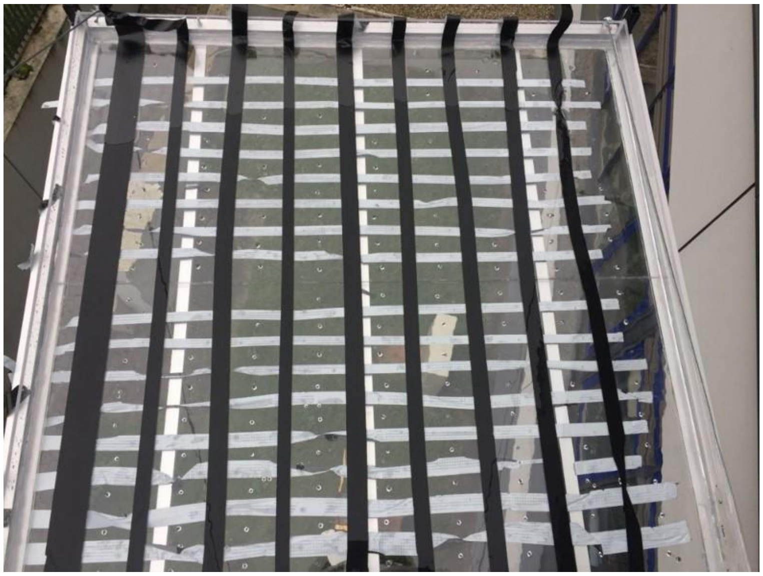

S2 Fig. Picture of the rain generator: The outer aluminum frame (1 x 1 x 0.05 m) was filled with water and raindrops formed at regularly arranged holes in the transparent bottom plate. The rainfall rate was varied by closing or opening a varying number of holes using adhesive tape (visible as black and silver tape stripes in the picture).
