# Supplementary material for: Rainfall as a driver for near-surface turbulence and air-water gas exchange in freshwater aquatic systems
Source: PLoS One. 2024 Mar 12;19(3):e0299998. doi: 10.1371/journal.pone.0299998 (PMC10931499; doi:10.1371/journal.pone.0299998)
Supplement: S3 Fig — Data were obtained from measurements with 1h resolution for the periods from 19-Nov-2019 to 09-Mar-2020 and from 01-Sep-2021 to 10-Dec-2021 (n = 81601). a. Rainfall rate frequencies, b. Rain events frequencies at different wind speed. (PDF) [file pone.0299998.s005.pdf]

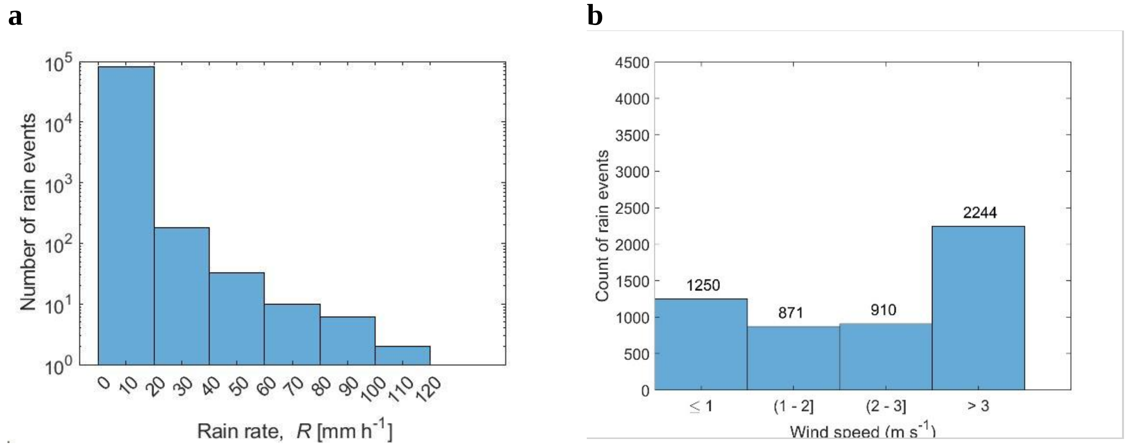

S3 Fig. Histogram of rain events collected by a meteorological station located on the water surface of Porce III reservoir, Colombia (6°54'12.6"N, 75°10'16.1"W). Data were obtained from measurements with 1h resolution for the periods from 19-Nov-2019 to 09-Mar-2020 and from 01-Sep-2021 to 10-Dec-2021 (n = 81601). **a.** Rainfall rate frequencies, **b.** Rain events frequencies at different wind speed.
