# Supplementary material for: Rainfall as a driver for near-surface turbulence and air-water gas exchange in freshwater aquatic systems
Source: PLoS One. 2024 Mar 12;19(3):e0299998. doi: 10.1371/journal.pone.0299998 (PMC10931499; doi:10.1371/journal.pone.0299998)
Supplement: S4 Fig — The slopes (in mm min-1) and regression statistics (the p-value is the significance of the slope applying the t-test, and n is the number of points used for the fit and t-test) reported in the text boxes are for the linear regressions (red solid lines) using all data (15 min of measurements). (PDF) [file pone.0299998.s006.pdf]

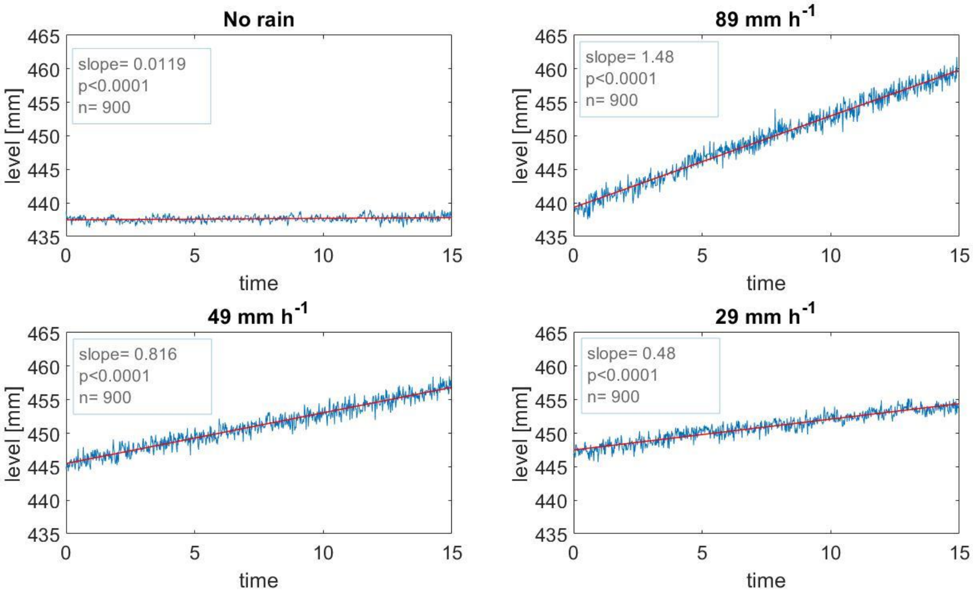

S4 Fig. Time series of pressure measured with the RBR sensor during an experimental series: without rain and for three different rain rates (see panel headings). The slopes (in mm min^{-1}) and regression statistics (the p-value is the significance of the slope applying the t-test, and *n* is the number of points used for the fit and t-test) reported in the text boxes are for the linear regressions (red solid lines) using all data (15 min of measurements).
