# Supplementary material for: Rainfall as a driver for near-surface turbulence and air-water gas exchange in freshwater aquatic systems
Source: PLoS One. 2024 Mar 12;19(3):e0299998. doi: 10.1371/journal.pone.0299998 (PMC10931499; doi:10.1371/journal.pone.0299998)
Supplement: S5 Fig — Red solid lines show linear regressions that were used for estimating the gas transfer velocity according to Eq (2). The slopes (in μmol L-1 min-1) in the text boxes are those of linear regressions using all data, the p-value is the significance of the slope applying the t-test, and n is the number of points used for the fit. (PDF) [file pone.0299998.s007.pdf]

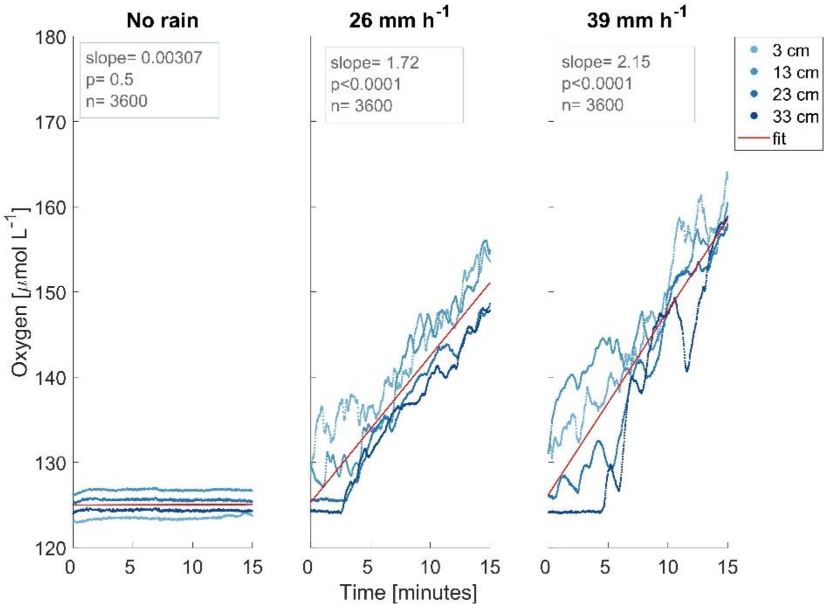

S5 Fig. Time series of dissolved oxygen concentration at different sampling depth in the aquarium (line color, see legend) observed without rain and for two different rain rates (see panel headings). Red solid lines show linear regressions that were used for estimating the gas transfer velocity according to Eq. (3). The slopes (in µmol L^{-1} min^{-1}) in the text boxes are those of linear regressions using all data, the p-value is the significance of the slope applying the t-test, and n is the number of points used for the fit.
