# Supplementary material for: Rainfall as a driver for near-surface turbulence and air-water gas exchange in freshwater aquatic systems
Source: PLoS One. 2024 Mar 12;19(3):e0299998. doi: 10.1371/journal.pone.0299998 (PMC10931499; doi:10.1371/journal.pone.0299998)
Supplement: S6 Fig — (PDF) [file pone.0299998.s008.pdf]

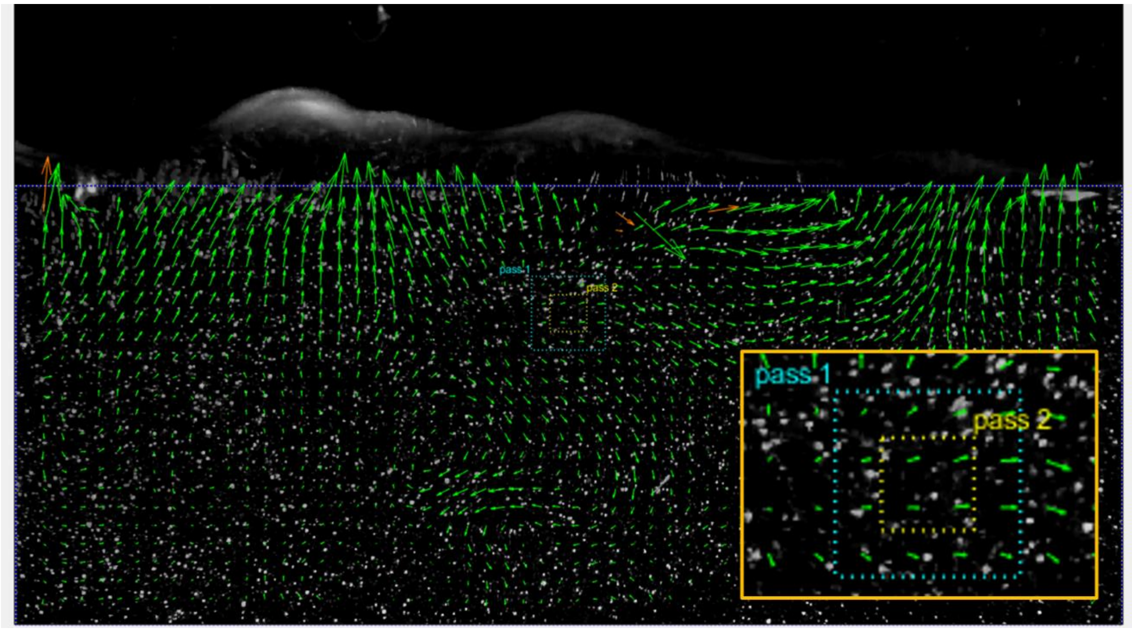

S6 Fig. Example of the analysis results in PIVlab of a pair pre-processed images: Region of interest (dark blue dashed line) extracted from the field of view; fluorescent particles in water (white points); the velocity vectors resulting from the analysis of a pair images (green arrows) and the interrogation areas from the two passes used in the data analysis (zoom at left-bottom in the yellow square).
