# Supplementary material for: Rainfall as a driver for near-surface turbulence and air-water gas exchange in freshwater aquatic systems
Source: PLoS One. 2024 Mar 12;19(3):e0299998. doi: 10.1371/journal.pone.0299998 (PMC10931499; doi:10.1371/journal.pone.0299998)
Supplement: S7 Fig — (PDF) [file pone.0299998.s009.pdf]

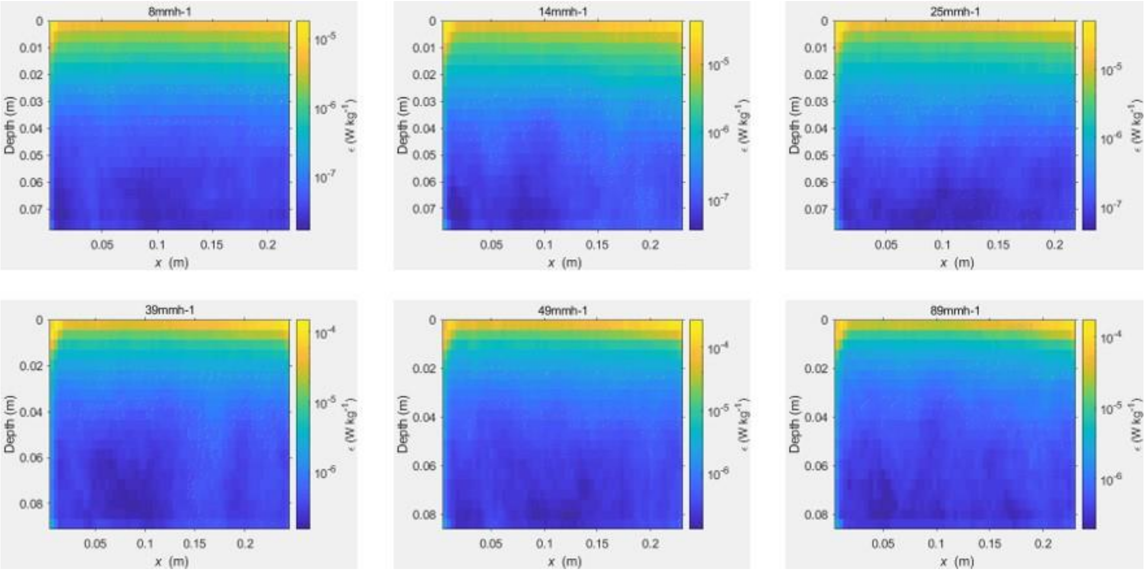


**S7 Fig.** Spatial distribution of the time-averaged turbulent dissipation rates ($\epsilon_{t\_avg}$) for six different rain rates (see panel titles).
