# Supplementary material for: Rainfall as a driver for near-surface turbulence and air-water gas exchange in freshwater aquatic systems
Source: PLoS One. 2024 Mar 12;19(3):e0299998. doi: 10.1371/journal.pone.0299998 (PMC10931499; doi:10.1371/journal.pone.0299998)
Supplement: S8 Fig — The solid lines show power-law fits according to the function shown in each legend. (PDF) [file pone.0299998.s010.pdf]

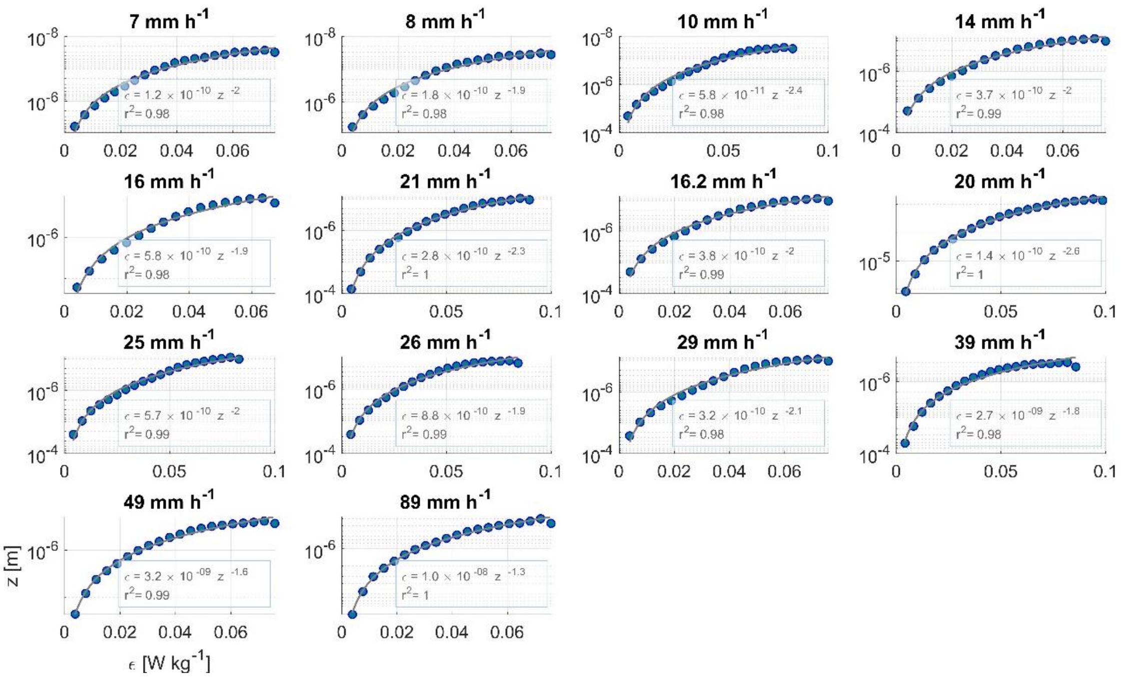

S8 Fig. Mean vertical profiles of dissipation rates of turbulent kinetic energy for all measured rain rates (filled symbols). The solid lines show power-law fits according to the function shown in each legend.
