# Supplementary material for: Rainfall as a driver for near-surface turbulence and air-water gas exchange in freshwater aquatic systems
Source: PLoS One. 2024 Mar 12;19(3):e0299998. doi: 10.1371/journal.pone.0299998 (PMC10931499; doi:10.1371/journal.pone.0299998)
Supplement: S9 Fig — Solid lines show linear regressions according to the equation shown in the legends. The best fit, which was chosen to estimate the empirical coefficient A is highlighted by the green bounding box. (PDF) [file pone.0299998.s011.pdf]

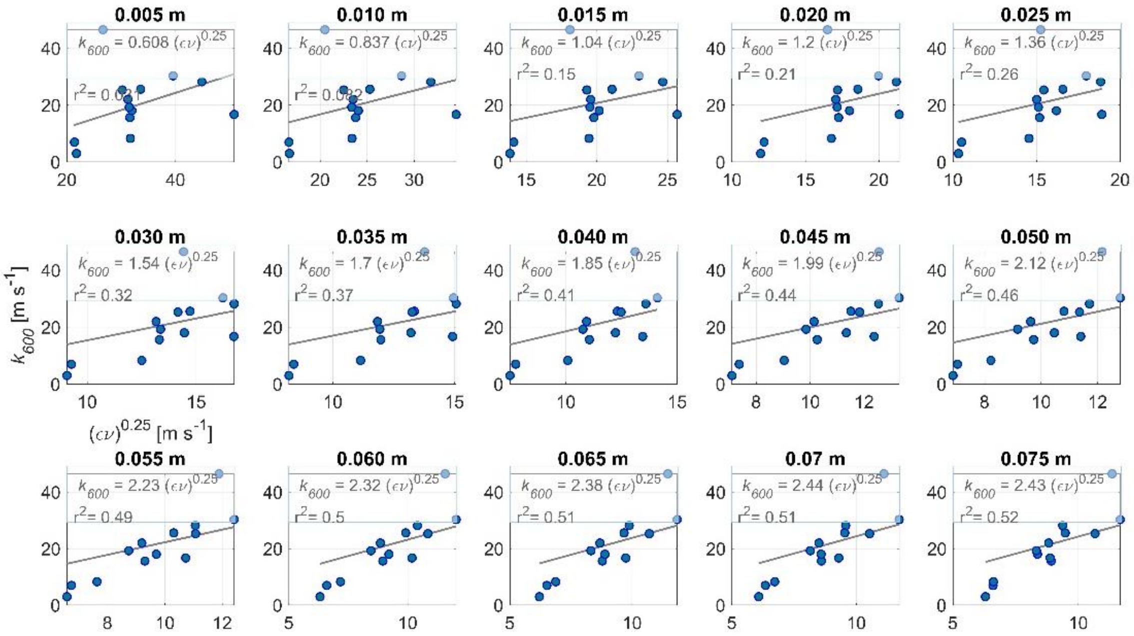

S9 Fig. Normalized gas transfer velocities k_{600} versus the surface renewal model (Eq. (13)) for different water depth at which dissipation rates (ϵ) were measured. Solid lines show linear regressions according to the equation shown in the legends. The best fit, which was chosen to estimate the empirical coefficient A is highlighted by the green bounding box.
