# Supplementary material for: Rainfall as a driver for near-surface turbulence and air-water gas exchange in freshwater aquatic systems
Source: PLoS One. 2024 Mar 12;19(3):e0299998. doi: 10.1371/journal.pone.0299998 (PMC10931499; doi:10.1371/journal.pone.0299998)
Supplement: S10 Fig — The solid line shows a polynomial fit according to the equation shown in the legend. (PDF) [file pone.0299998.s012.pdf]

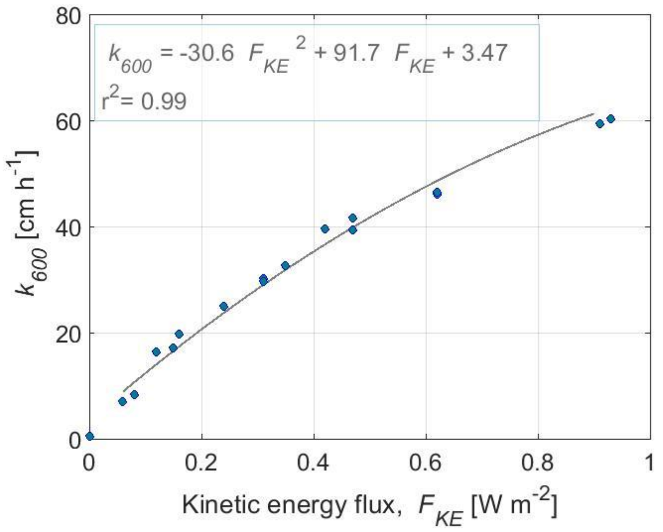

S10 Fig. Normalized gas transfer velocity k_{600} as a function of the kinetic energy flux of rain reported in Ho et al. (1997) (their Table 1). The solid line shows a polynomial fit according to the equation shown in the legend.

References

Ho DT, Bliven LF, Wanninkhof R, Schlosser P. The effect of rain on air-water gas exchange. Tellus B: Chemical and Physical Meteorology. 1997;49: 149–158. doi:10.3402/tellusb.v49i2.15957
