# Supplementary material for: Rainfall as a driver for near-surface turbulence and air-water gas exchange in freshwater aquatic systems
Source: PLoS One. 2024 Mar 12;19(3):e0299998. doi: 10.1371/journal.pone.0299998 (PMC10931499; doi:10.1371/journal.pone.0299998)
Supplement: S11 Fig — The solid line shows a polynomial fit according the equation shown in the legend. In the legend, the values in parenthesis report the averaged drop size for freshwater experiments corresponding to the results of Ho et al. (2000) and saltwater experiments correspond to the results of Zappa et al. (2009), in which there was a broad drop size distribution (DSD). (PDF) [file pone.0299998.s013.pdf]

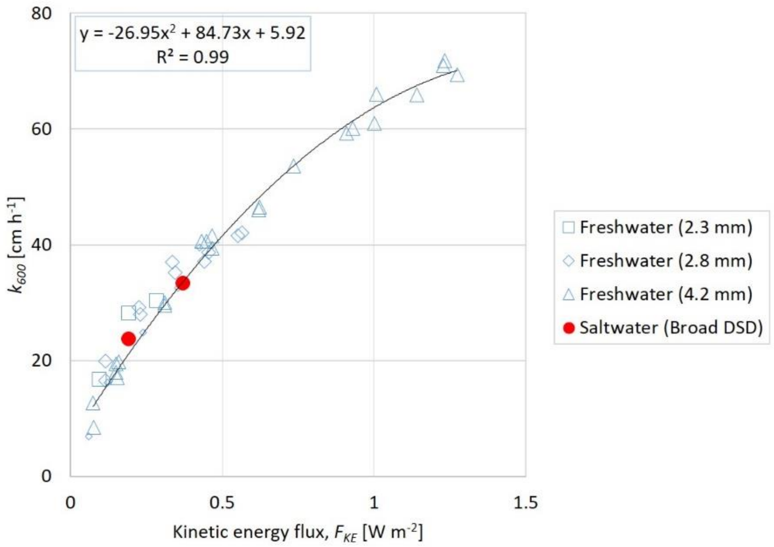

S11 Fig. Normalized gas transfer velocity k_{600} as a function of the kinetic energy flux of rain reported in Ho et al. (2000) and Zappa et al. (2009) (taken from Fig 8 in Zappa et al. (2009)). The solid line shows a polynomial fit according the equation shown in the legend. In the legend, the values in parenthesis report the averaged drop size for freshwater experiments corresponding to the results of Ho et al. (2000) and saltwater experiments correspond to the results of Zappa et al. (2009), in which there was a broad drop size distribution (DSD).

References

Ho DT, Asher WE, Bliven LF, Schlosser P, Gordan EL. On mechanisms of rain-induced air-water gas exchange. Journal of Geophysical Research: Oceans. 2000;105: 24045–24057. doi:10.1029/1999jc000280

Zappa CJ, Ho DT, McGillis WR, Banner ML, Dacey JWH, Bliven LF, et al. Rain-induced turbulence and air-sea gas transfer. Journal of Geophysical Research: Oceans. 2009;114: 1–17. doi:10.1029/2008JC005008
