# Supplementary material for: Rainfall as a driver for near-surface turbulence and air-water gas exchange in freshwater aquatic systems
Source: PLoS One. 2024 Mar 12;19(3):e0299998. doi: 10.1371/journal.pone.0299998 (PMC10931499; doi:10.1371/journal.pone.0299998)
Supplement: Attachment — Submitted filename: Response_to_Reviewers-final.docx [file pone.0299998.s014.docx]

# Response to comments on the revised manuscript

Title: Rainfall as a Driver for Near-Surface Turbulence and Air-Water Gas Exchange in Freshwater Aquatic Systems

## Response to editors

Please ensure that your manuscript meets PLOS ONE's style requirements, including those for file naming. The PLOS ONE style templates can be found at https://journals.plos.org/plosone/s/file?id=wjVg/PLOSOne_formatting_sample_main_body.pdf and <https://journals.plos.org/plosone/s/file?id=ba62/PLOSOne_formatting_sample_title_authors_affiliations.pdf>

This submission meets Plos One’s style requirements based on the templates and on-line guides.

Thank you for stating the following financial disclosure:

"This study was partially supported by the German Research Foundation (DFG), project number LO 1150/12-1 (AL) and RO 5921/1-1 (LR). <https://www.dfg.de/en/>

 EBB. was funded by the following programs:

- Research Grants - Short-Term Grants, 2019 (57440917) of the German Academic Exchange Service (DAAD), <https://www.daad.co/es>

- Scholarship Program No. 757 - National Doctorates of the Ministry of Science, Technology and Innovation of Colombia, <https://minciencias.gov.co/>

- the Call for Teaching and Student Mobility of 2019-2021 of the Facultad de Minas of the Universidad Nacional de Colombia, <https://minas.medellin.unal.edu.co/>"

After verification, it has been established that these statements are correct and complete. No changes are necessary here.

Please state what role the funders took in the study.  If the funders had no role, please state: "The funders had no role in study design, data collection and analysis, decision to publish, or preparation of the manuscript." If this statement is not correct you must amend it as needed.

Please include this amended Role of Funder statement in your cover letter; we will change the online submission form on your behalf.

Certainly, the funders had no role in study design, data collection and analysis, decision to publish, or preparation of the manuscript.

Please ensure that you refer to Figure 7 in your text as, if accepted, production will need this reference to link the reader to the figure.

After verification, it has been established that Figure 7 is cited in the original submitted manuscript in lines 499 and 513. In the revised version, its reference can be found in lines 502 and 517.

Please upload a copy of Figures 8 & 9, to which you refer in your text on pages 24 and 20. If the figure is no longer to be included as part of the submission please remove all reference to it within the text.

The mentions of Figures 8 and 9 on pages 20 and 24 are connected to the citation of the work of Zappa et al. (2009), where those figures are presented. To prevent any potential confusion, we now only refer to the study itself, and not explicitly to the figures within.

Please review your reference list to ensure that it is complete and correct. If you have cited papers that have been retracted, please include the rationale for doing so in the manuscript text, or remove these references and replace them with relevant current references. Any changes to the reference list should be mentioned in the rebuttal letter that accompanies your revised manuscript. If you need to cite a retracted article, indicate the article’s retracted status in the References list and also include a citation and full reference for the retraction notice.

No retracted articles were cited in our manuscript. Two references [35] and [36] were added to the manuscript.

## Response to Reviewer #1

The abstract part has to introduce the main objective and problem statement for why the study important. It needs modification.

The abstract has been revised and now includes the main objective and the problem statement. L XX to XX.

On page 4 line number 80 introduction part, you mention overlooking low to moderate of rainfall intensities. Is there any consideration in your study to identify intensity of rainfall?

Indeed, for selecting the intensity of rainfall for our study, we specifically considered the most typical rainfall rates under actual field conditions at the tropical reservoir Porce III. To better understand this approach, it is helpful to highlight that our study is an integral component of a doctoral thesis that included a field phase conducted at this location.

In the line 165 of the revised version of the manuscript, the Figure S3 (S3 Fig) was referred to show these observations in histograms.

On page 7 line number 132 you mentioned the inquire additional measurements for rain rates, size and fall velocity of raindrops. Have overcome the significant changes of size and fall velocity in your experiment?

Actually, the line 132 in the original manuscript means that, additional to the main measurements of gas transfer velocity and turbulence, other measurements corresponding to rain rates and the size and fall velocity of raindrops were performed. The original sentence was adjusted to avoid future confusion and now it is clearer that these measurements are part of the present study (see L 136).

Certainty one limitation of our study is the lack of direct measurements of the drops (drop sizes and drop velocity) during all experimental runs. Fortunately, regarding the terminal velocity was one of the main characteristics of our experiment and 20 m height from the aquarium to the rain generation is more than enough to reach terminal velocity (lines 131-132, 236, 546 in the revised version). Therefore, no significant changes are expected in the fall velocity among the experimental runs.

On the other hand, it was difficult to measure the drop size distribution and we consider this aspect as a limitation of our study. As mentioned in the methods, we used several configurations to cover the range of different rain rates. These configurations, in part, consisted of opening holes with different sizes (at least 2) in the rain generator. Only the largest drops were measured (coming from 0.8 mm open holes). The smaller holes corresponded to tape pierced with a 0.4 mm needle, where the drop sizes where not measured. Therefore, we assumed a range of drops were the upper limit is the measured ones produced from 0.8 mm holes and the lower limit is the diameters obtained in previous studies where 0.4 mm needles were used to form drops (L 232-235 of the revised version).

1. On page 8 line number 159, when you adjusted the number of open holes to target a range of rain rates between 7 and 90 mm h-1, how you identified it’s the most frequent rain rates?

As mentioned earlier, we considered the most typical rain rates under actual field conditions at the tropical reservoir Porce III and consulted relevant literature:

(Gaál et al., 2014) discuss a series of references regarding the threshold for identifying intense warm-season storms with a convective character. The cited range in Gaál et al. (2014) is between 15 and 50 mm h^-1^ as the threshold to consider heavy rainfall in temperate environments.

(Rooney et al., 2018) report a measured range of 0.2 to 500 mm h^-1^ in a tropical African lake.

(Yakubu et al., 2016) classify storms in a tropical location in Malaysia as typical very high-intensity storms and typical medium-intensity storms. In the case of very high intensity, the rain distribution over 100 minutes ranges from less than 10 mm h^-1^, with a peak around 130 mm h^-1^, and a dominant intensity of around 40 mm h^-1^. For medium-intensity storms, the highest peak is at 70 mm h^-1^, with a mean of about 15 mm h^-1^.

Based on these references, additional to our own data measured at Porce III reservoir, we consider that a range between 7 and 90 mm h^-1^ well represents the typical intensities occurring in nature.

## It's important to note that the range of 7 to 90 mm h^-1^ was determined based on our experimental setups. Initially, we did not precisely set this range as our target. The initial target range was approximately 5 to 100 mm h^-1^, and throughout the experiment, we measured the intensities to ultimately identify the 14 specific rain rates corresponding to the rainmaker configuration in each run.

The text in the manuscript was revised to provide a clearer explanation for this range, including the references above (L 165-166 of the revised version).

Cited references

Gaál L, Molnar P, Szolgay J. Selection of intense rainfall events based on intensity thresholds and lightning data in Switzerland. Hydrol Earth Syst Sci. 2014;18: 1561–1573. doi:10.5194/hess-18-1561-2014

Rooney GG, van Lipzig N, Thiery W. Estimating the effect of rainfall on the surface temperature of a tropical lake. Hydrol Earth Syst Sci. 2018;22: 6357–6369. doi:10.5194/hess-22-6357-2018

## Yakubu ML, Yusop Z, Fulazzaky MA. The influence of rain intensity on raindrop diameter and the kinetics of tropical rainfall: case study of Skudai, Malaysia. Hydrological Sciences Journal. 2016;61: 944–951. doi:10.1080/02626667.2014.934251

## Response to Reviewer #2

1. Authors are encouraged to engage in a comprehensive discussion of the outcomes, elucidating their potential interpretations in light of prior investigations and the underlying hypotheses. Furthermore, it is essential to deliberate upon the broader implications of the findings and their significance within a wider framework. Additionally, it is advisable to emphasize potential avenues for future research.

We respond to this comment by reviewing the three points made by the reviewer:

Authors are encouraged to engage in a comprehensive discussion of the outcomes, elucidating their potential interpretations in light of prior investigations and the underlying hypotheses

In relation to this point, we have carefully reviewed the discussion and identified that the primary findings were deliberated upon as the outcome of an exhaustive literature review, considering key studies. It is pertinent to note that this particular subject has not had widespread attention, with only a limited number of researchers having contributed to the existing body of knowledge. Among the available publications, only a few are dedicated to exploring the turbulence induced by rain. Consequently, due to the lack of references, the discussion of results relies on a limited pool of published papers. For a more detailed understanding of how we approached the discussion of results, please refer to the summarized paragraphs throughout the discussion section:

- In paragraph 1: Under the underlying hypothesis that the mechanism by which the gas transfer velocity increases is turbulence, a scaling based on direct estimates of turbulence is good for describing the proposed relationship between gas transfer rate and rain rate. We discuss the idea of a systematic increase in turbulence as an effect of rain rate was not previously reported, despite the effort of previous studies [10,25,28]. We hypothesize that this positive result was obtained due to the use of a wide range of rainfall rates, from low to heavy intensities and the consideration of 14 observations, being an advantage compared to other studies.
- In paragraph 2: we analyze the magnitude of turbulence in comparison with previous studies [10] [11]. At first look, it appears that our results underestimate the turbulence. Assuming this is the case, the premise is that this could occur due to the insufficient resolution of our PIV setup. We then formulated a scaling analysis proposed by [49] and estimated that we resolved about 75% of the total dissipation rate, which corresponds to our initial underlying hypothesis that PIV is a suitable method to investigate our research problem.
- In paragraph 3: in addition to the analysis of the scales, we applied a supporting method during the experiment. Dissipation rates were estimated by the inertial subrange method using acoustic Doppler velocimeter (ADV) data. The results showed that no large differences were observed between the PIV and ADV data.
- In paragraph 4: summary of the discussion above. Our results suggest that the difference with previous studies was not an underestimation of turbulence, but we hypothesize that other factors may influence the difference between our study and others. One important factor is that other studies combine turbulence from other mechanisms rather than raindrops, which may become dominant over rain-generated turbulence. An important point arises here as our results come from the study of rain in isolation, our scaling should be applicable to rain and low wind conditions.

Furthermore, it is essential to deliberate upon the broader implications of the findings and their significance within a wider framework

Certainly, the reviewer makes an excellent point here. Actually, in the first version of the manuscript, some of the ideas regarding broader implications were placed in the conclusion section. In the new version, in response to the reviewer's suggestion, a section entitled Broader Implications was added as a subsection of the discussion, including several references to support the statements [22,23] [25] [6] [24] [53–55]. This section seeks to elucidate the relevance of our findings on air-water gas fluxes and their potential to contribute to a more complete understanding of greenhouse gas dynamics in aquatic ecosystems, especially in tropical inland waters.

Additionally, it is advisable to emphasize potential avenues for future research.

As a complement to the Broader implications section, we have included some lines on the recommendation of future research, pointing out that future research may cover the interaction of several turbulence generation mechanisms, simultaneously measure raindrops size and velocity and validate our findings under field conditions.
